# Development and validation of new screening tool for predicting dementia risk in community-dwelling older Japanese adults

**DOI:** 10.1186/s12967-021-03121-9

**Published:** 2021-10-26

**Authors:** Keitaro Makino, Sangyoon Lee, Seongryu Bae, Ippei Chiba, Kenji Harada, Osamu Katayama, Yohei Shinkai, Hiroyuki Shimada

**Affiliations:** 1 Department of Preventive Gerontology, Center for Gerontology and Social Science, National Center for Geriatrics and Gerontology, 7-430 Morioka-cho, Obu, Aichi 474-8511 Japan; 2 Japan Society for the Promotion of Science, Chiyoda-ku, Tokyo, Japan; 3 Center for Gerontology and Social Science, National Center for Geriatrics and Gerontology, Obu, Aichi Japan

**Keywords:** Dementia prevention, Decision-tree, Machine learning, Risk prediction

## Abstract

**Background:**

Established clinical assessments for detecting dementia risk often require time, cost, and face-to-face meetings. We aimed to develop a Simplified Telephone Assessment for Dementia risk (STAD) (a new screening tool utilizing telephonic interviews to predict dementia risk) and examine the predictive validity of the STAD for the incidence of dementia.

**Methods:**

We developed STAD based on a combination of literature review, statistical analysis, and expert opinion. We selected 12 binary questions on subjective cognitive complaints, depressive symptoms, and lifestyle activities. In the validation study, we used STAD for 4298 community-dwelling older adults and observed the incidence of dementia during the 24-month follow-up period. The total score of STAD ranging from 0 to 12 was calculated, and the cut-off point for dementia incidence was determined using the Youden index. The survival rate of dementia incidence according to the cut-off points was determined. Furthermore, we used a decision-tree model (classification and regression tree, CART) to enhance the predictive ability of STAD for dementia risk screening.

**Results:**

The cut-off point of STAD was set at 4/5. Participants scoring ≥ 5 points showed a significantly higher risk of dementia than those scoring ≤ 4 points, even after adjusting for covariates (hazard ratio [95% confidence interval], 2.67 [1.40–5.08]). A decision tree model using the CART algorithm was constructed using 12 nodes with three STAD items. It showed better performance for dementia prediction in terms of accuracy and specificity as compared to the logistic regression model, although its sensitivity was worse than the logistic regression model.

**Conclusions:**

We developed a 12-item questionnaire, STAD, as a screening tool to predict dementia risk utilizing telephonic interviews and confirmed its predictive validity. Our findings might provide useful information for early screening of dementia risk and enable bridging between community and clinical settings. Additionally, STAD could be employed without face-to-face meetings in a short time; therefore, it may be a suitable screening tool for community-dwelling older adults who have negative attitudes toward clinical examination or are non-adherent to follow-up assessments in clinical trials.

**Supplementary Information:**

The online version contains supplementary material available at 10.1186/s12967-021-03121-9.

## Background

The prevalence of dementia is rapidly increasing along with an aging global population. According to predictions, the total number of people with dementia will reach 82 million in 2030 and 152 million in 2050 [1], which will pose heavy social and economic burden. Therefore, early screening of dementia risk is needed to promote timely preventive strategies.

Considering screening tools for dementia risk, several assessment methods with high accuracies, such as brain scans, blood sampling, or neuropsychological tests, are established. However, these clinical examinations are relatively expensive and require face-to-face meetings; thus, they may be unsuitable for primary screening of large populations in community settings. Recently, some non-face-to-face risk screening tools, including computerized self-assessment [2] and telephonic interviews [3], have been proposed. However, most of them require computer literacy, family cooperation, or expert judgment in risk assessment, and there are certain restrictions in their application range. Although there are several telephone-based neurocognitive tests proposed in previous studies [3], they include questions to test cognitive functions directly, and their acceptability in the first telephonic interview is unclear. An earlier systematic review showed that non-participants of health checkups tend to have attendance barriers, including time constraints or aversion to preventive medicine [4]; therefore, a fast and simple dementia risk screening method that is acceptable to older adults with negative attitudes toward participation in health checkups or clinical examinations should be developed. Additionally, in research activities, long-term surveys or intervention studies requiring face-to-face assessment are often accompanied by dropout of subjects during follow-up. Thus, alternative methods to assess the minimum necessary outcome among subjects who cannot participate in venue-based assessments are required for accurate investigation.

Considering dementia risk assessment methods that could be conducted through non-face-to-face settings without medical workers, previous studies have demonstrated that some simplified questionnaires to assess subjective cognitive complaints [5], depressive symptoms [6], and engagement in lifestyle activities [7] could predict dementia risk from longitudinal analyses. Therefore, we hypothesized that a simplified telephone interview, including an appropriate combination of simplified questionnaires, could be useful to predict dementia risk without face-to-face meetings, subject burden, and expert judgment among community settings.

Recently, machine learning methods that can learn nonlinear interactions iteratively from large samples using computer algorithms have been applied in various fields, such as disease risk assessment and prediction [8]. Particularly, decision-tree analysis using the classification and regression tree (CART) algorithm provides a rather intuitive diagram to represent risk prediction without complicated calculations [9]. Thus, the CART method has been used in many areas for decision-making purposes to develop models that can classify subjects into various risk categories [10]. We identified a few previous studies that have examined the usefulness of predictive models, including non-biological risk factors for dementia, using a decision-tree analysis [11–13]. However, the usefulness of the decision-tree model that included easily obtainable risk factors in old age for predicting the risk of dementia is unknown.

Therefore, we aimed to develop a Simplified Telephone Assessment for Dementia risk (STAD), a screening tool to predict dementia risk that utilizes telephonic interviews and examine the predictive validity of the STAD for dementia incidence using a basic regression model and a decision-tree model.

## Methods

### Study settings and participants

This cohort study included community-dwelling older adults enrolled from a sub-cohort of the National Center for Geriatrics and Gerontology-Study of Geriatric Syndromes (NCGG-SGS). NCGG-SGS is a Japanese national cohort study; its primary goal was to establish a screening system for geriatric syndromes and validate evidence-based preventive interventions [14]. Individuals aged ≥ 65 years living in Obu City that were not hospitalized, not in residential care, not certified by the national long-term care insurance system (LTCI) as having a functional disability, or not participating in another study (n = 14,313) were sent an invitation letter to participate in a baseline assessment. We assessed 5104 individuals at baseline (August 2011 to February 2012). We applied the following exclusion criteria: (i) those with a history of dementia (n = 140), (ii) those with suspected dementia at baseline based on Mini-Mental State Examination (MMSE) score < 21 [15] (n = 146); (iii) those with Parkinson’s disease (n = 21), (iv) those with depression (n = 130), (v) those dependent on others for basic activities of daily life, such as eating, bathing, grooming, walking, and stair-climbing (n = 20), (vi) those with a functional disability based on the LTCI system (n = 74), and (vii) those with missing data for these criteria or questionnaires at baseline assessment (n = 95). After exclusion, 4478 cognitively intact participants were identified. During the follow-up period, participants who were unable to confirm public health insurance affiliations based on the Japanese National Health Insurance and Later-Stage Medical Care Systems (n = 180) were excluded from the analysis. Therefore, 4298 participants were included in the final longitudinal analysis.

All baseline assessments were performed as health check-ups by well-trained nurses and study assistants in community centers. All staff received training from the authors on the protocols for administering the assessments before the study began. During the follow-up period, we collected medical records of Japanese public health insurance to identify the incidence of dementia. Data from medical records were collected from the local government on a monthly basis for 24 months.

### Ethics approval

The study protocol was developed in accordance with the Declaration of Helsinki and was approved by the ethics committee of the National Center for Geriatrics and Gerontology. Prior to participation in the study, written informed consent was obtained from all participants.

### Development of STAD and screening of dementia risk

STAD was developed based on a combination of literature review, statistical analysis, and expert opinion. First, we created a questionnaire on dementia risk consisting of 30 “yes”/“no” questions, which included subjective memory complaints, depressive symptoms, functions in daily living, and lifestyle activities based on a literature review. We then assessed the participants using a questionnaire at baseline and followed them up for 24-months to detect dementia incidence. Second, we examined the relationships between each item of the questionnaire and dementia incidence using Cox proportional hazards regression analysis, and 23 significant predictors (after adjusting for age and sex) were identified as candidate items of STAD (Additional file 1: Table S1). Third, a panel of five experts (geriatrics and health science specialists) examined the content validity of the candidate items using the content validity index (CVI) [16]. The content validity was assessed in terms of clarity, concreteness, essentiality, and importance for the prediction of dementia risk using a 4-point Likert scale (e.g., 1, not clear; 2, not very clear, 3, somewhat clear; and 4, very clear). The CVI of each item was calculated as the number of experts giving a rating of 3 or 4 divided by the total number of experts [16]. The results range from 0 to 1 with the following interpretation, > 0.79, the item was relevant; 0.70–0.79, the item needed revision; and < 0.70, the item was eliminated [16] (Additional file 1: Table S2). Finally, 11 items were eliminated and the remaining 12 were included in the STAD: (1) Do you forget where you have left things more than you used to? [5], (2) Do other people find you forgetful? [5], (3) Do you find yourself not knowing today’s date? [17], (4) Have you dropped many of your activities and interests? [18], (5) Do you often get bored? [18], (6) Do you feel helpless? [18], (7) Do you prefer to stay at home, rather than going out and doing new things? [18], (8) In the last 2 weeks have you felt tired without a reason? [17], (9) Do you go out less frequently compared to last year? [17], (10) Do you engage in low levels of physical exercise aimed at health at least five times a week? [19], (11) Do you use maps to go to unfamiliar places? [20], and (12) Do you engage in cognitive stimulation, such as board games and learning [20]? Participants were assessed for the risk of dementia using the STAD. The total score (0–12) was calculated by adding the number of risks at baseline.

### Observation of dementia incidence

All individuals aged ≥ 65 years had one of the following types of public health insurance in Japan: “Employees’ Health Insurance,” “Japanese National Health Insurance,” or “Later-Stage Medical Care System” [21, 22]. Individuals aged 65–74 years enroll in either one of the Employees’ Health Insurance (health insurance for employed individuals aged < 75 years) or the Japanese National Health Insurance (national health insurance for unemployed and self-employed individuals aged < 75 years). When they reach 75 years, they are automatically switched to Later-Stage Medical Care System (health care for individuals aged ≥ 75 years). We checked the Japanese National Health Insurance and Later-Stage Medical Care Systems for data regarding newly reported cases of dementia and the date of diagnosis every month. We defined “the incidence of dementia” as a new diagnosis of dementia during the 24-month follow-up period, but not at baseline. The diagnosis of dementia was made by medical doctors in medical facilities according to the International Classification of Diseases-10 [23].

### Assessments of potential confounding factors

As covariates, age, sex, educational attainment, and comorbidities (hypertension, hyperlipidemia, diabetes mellitus, and heart disease) were assessed through face-to-face interviews at baseline. We also included drinking and smoking habits (current vs. former/never), slow gait speed, physical inactivity, living arrangements (living alone or cohabiting), and global cognitive function at baseline as covariates. The gait speed was measured over a 2.4-m distance, and the mean gait speed of five trials of < 1.0 m/s was defined as slow gait speed [24]. The physical inactivity was evaluated by asking the following: (1) “Do you engage in more than moderate levels of physical exercise or sports aimed at health?” and (2) “Do you engage in low levels of physical exercise aimed at health?” Participants who responded “no” to both questions were classified as inactive [14]. The global cognitive function was assessed using the MMSE; the MMSE scores ranged from 0 to 30, with higher scores indicating better cognitive performance [25].

### Statistical analyses

To begin with, we compared baseline characteristics between participants with and without dementia incidence using Student’s t-test for continuous variables and χ^2^ test for categorical variables. And then, for model construction and validation, we randomly divided the dataset into training and test datasets in a 6:4 ratio.

First, in the training dataset, the optimal cut-off points of the STAD score that best discriminated participants who developed and did not develop dementia were identified using the Youden Index [26]. In the test dataset, the cumulative survival rate of the incidence of dementia during the 24-month follow-up according to the cut-off points was calculated using Kaplan–Meier curves, and intergroup differences were estimated using a log-rank test. Additionally, a Cox proportional hazards regression analysis was conducted to examine the predictive validity of STAD cut-off points for the prediction of dementia incidence. The hazard ratios (HRs) were calculated with 95% confidence intervals (CIs) for the risk of dementia.

Second, we used a decision-tree model to enhance the predictive ability of STAD for dementia risk screening. We performed a decision-tree analysis using the CART algorithm to identify the optimal and minimum combination of STAD items for predicting the risk of dementia in the training dataset. The CART algorithm is based on recursive partitioning analysis, and the aim is to develop prediction rules by constructing binary trees. For this analysis, the Gini index [27] was used as the splitting criterion, which characterized the impurity of a sample set, and the maximum tree depth was set to 3. Additionally, the synthetic minority oversampling technique (SMOTE) [28] was applied to solve the problem of imbalanced data in the dementia status (incidence rate of dementia was only 2.2%), as some supervised algorithms showed worse performance with unbalanced datasets.

Third, a logistic regression model using cut-off points of the STAD score was also created as a benchmark to evaluate the decision-tree model. In the test dataset, we identified the model performance of the decision-tree model and logistic regression model using areas under the curve (AUC) based on the receiver operating characteristic (ROC) analysis, accuracy, sensitivity, and specificity. All analyses were performed using IBM SPSS Statistics 25 and IBM SPSS Modeler 18 (IBM Japan, Tokyo, Japan). The level of statistical significance was set at *P* < 0.05.

## Results

### Participant characteristics

Of the 4298 participants enrolled in our 24-month follow-up study, 93 (2.2%) were newly diagnosed with dementia. The differences in baseline characteristics between participants with and without dementia are shown in Table 1. Participants who developed dementia were significantly older (*P* < 0.001), more often female (*P* = 0.025), less educated (*P* < 0.001), had higher prevalence of heart disease (*P* = 0.039) and slow gait (*P* < 0.001), and lower MMSE scores (*P* < 0.001) than those who did not develop dementia.

**Table 1:** Baseline characteristics according to the incidence of dementia in all participants

	Overall *n* = 4,298	Participants without dementia incidence *n* = 4205	Participants with dementia incidence *n* = 93	*P-*value*
Age (years)	71.9 ± 5.4	71.8 ± 5.3	76.9 ± 6.1	< 0.001
Sex (female, %)	2139 (49.8)	2082 (49.5)	57 (61.3)	0.025
Education (years)	11.4 ± 2.5	11.4 ± 2.5	10.5 ± 2.7	< 0.001
Hypertension (n, %)	2000 (46.5)	1956 (46.5)	44 (47.3)	0.879
Heart disease (n, %)	723 (16.8)	700 (16.6)	23 (24.7)	0.039
Diabetes mellitus (n, %)	582 (13.5)	568 (13.5)	14 (15.1)	0.666
Hyperlipidemia (n, %)	1769 (41.2)	1736 (41.3)	33 (35.5)	0.261
Drinking habit (n, %)	2003 (46.6)	1966 (46.8)	37 (39.8)	0.183
Smoking habit (n, %)	429 (10.0)	419 (10.0)	10 (10.8)	0.802
Slow gait speed (n, %)	398 (9.3)	373 (8.9)	25 (26.9)	< 0.001
Living alone (n, %)	400 (9.3)	388 (9.2)	12 (12.9)	0.228
Physical inactivity (n, %)	1246 (29.0)	1,213 (28.8)	33 (35.5)	0.163
MMSE (score)	26.5 ± 2.4	26.5 ± 2.4	24.9 ± 2.4	< 0.001

The data are expressed as the mean ± standard deviation or numbers (%)

*MMSE* mini-mental state examination

*Based on Student’s t-test for continuous variables and χ^2^ tests for categorical variables

### Cut-off points and predictive validity of STAD

**Fig. 1:**
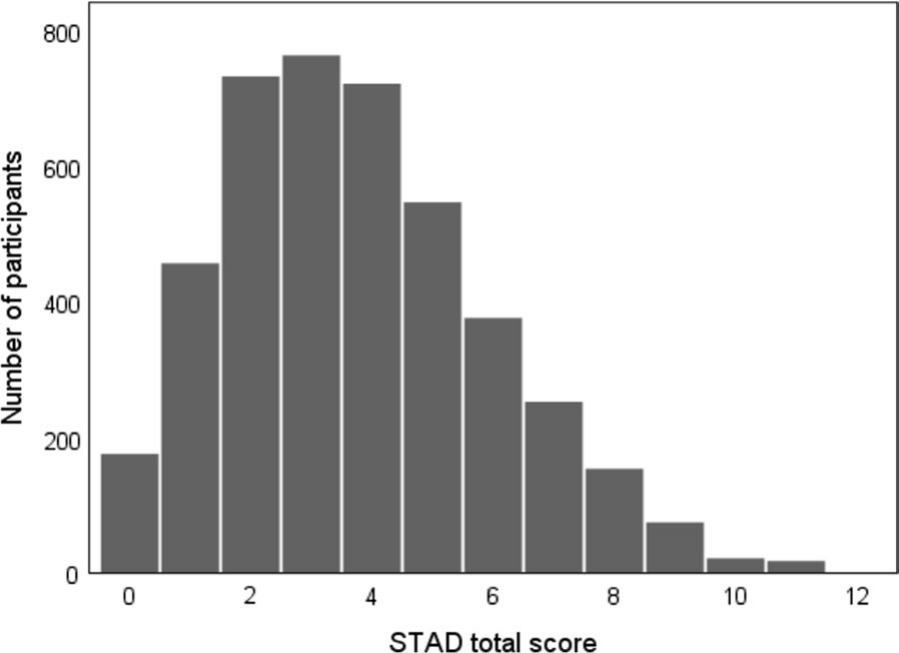
Histogram of the STAD score in all participants

The mean STAD score in all participants was 3.8 ± 2.2 (Fig. 1). The differences in STAD assessment results between participants with and without dementia are shown in Table 2. There were significant differences in the applicability rate of STAD items, except for questions 7 and 10 (*P* < 0.05) and STAD total scores (*P* < 0.001) between participants with and without dementia.

**Table 2:** Comparison of STAD assessment results according to dementia incidence in all participants

	Overall *n* = 4298	Participants without dementia incidence *n* = 4205	Participants with dementia incidence *n* = 93	*P-*values*
STAD items				
(1) Do you forget where you have left things more than you used to? (yes, %)	2463 (57.5)	2392 (56.9)	71 (76.3)	< 0.001
(2) Do other people find you forgetful? (yes, %)	840 (19.5)	808 (19.2)	32 (34.4)	< 0.001
(3) Do you find yourself not knowing today’s date? (yes, %)	1038 (24.2)	997 (23.7)	41 (44.1)	< 0.001
(4) Have you dropped many of your activities and interests? (yes, %)	1022 (23.8)	987 (23.5)	35 (37.6)	0.002
(5) Do you often get bored? (yes, %)	620 (14.4)	599 (14.2)	21 (22.6)	0.024
(6) Do you feel helpless? (yes, %)	1207 (28.1)	1163 (27.7)	44 (47.3)	< 0.001
(7) Do you prefer to stay at home, rather than going out and doing new things? (yes, %)	1420 (33.0)	1381 (32.8)	39 (41.9)	0.065
(8) In the last 2 weeks have you felt tired without a reason? (yes, %)	541 (12.6)	517 (12.3)	24 (25.8)	< 0.001
(9) Do you go out less frequently compared to last year? (yes, %)	606 (14.1)	583 (13.9)	23 (24.7)	0.003
(10) Do you engage in low levels of physical exercise aimed at health at least five times a week? (no, %)	2707 (63.0)	2640 (62.8)	67 (72.0)	0.067
(11) Do you use maps to go to unfamiliar places? (no, %)	1575 (36.6)	1521 (36.2)	54 (58.1)	< 0.001
(12) Do you engage in cognitive stimulation such as board games and learning? (no, %)	2163 (50.3)	2105 (50.1)	58 (62.4)	0.019
STAD total score (points)	3.8 ± 2.2	3.7 ± 2.2	5.5 ± 2.3	< 0.001

The data are expressed as the mean ± standard deviation or numbers (%)

*STAD* simplified telephone assessment for dementia risk

*Based on Student’s t-test for continuous variables and χ^2^ tests for categorical variables

**Fig. 2:**
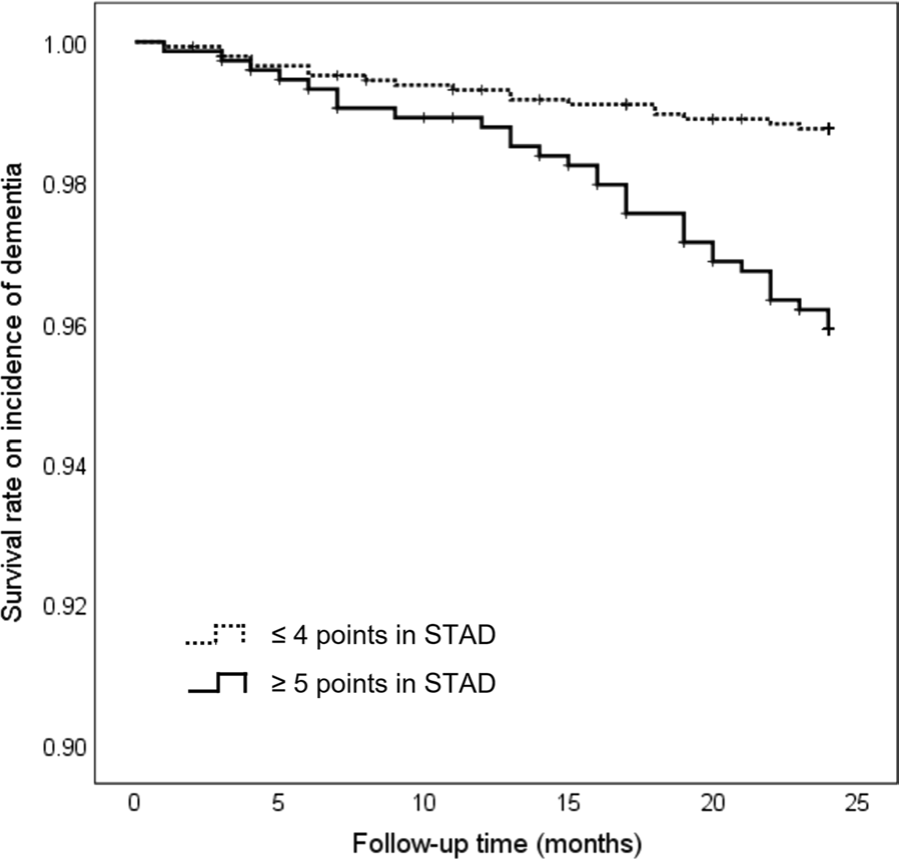
Cumulative survival rate on dementia incidence according to the STAD score in the test dataset

The optimal cut-off points of the STAD score for prediction of dementia incidence were identified as 4/5 points using the Youden Index. Of the 1750 participants included in the test dataset, 618 (35.3%) scored ≥ 5 points and they were significantly older (P < 0.001), more often female (P < 0.001), less educated (P < 0.001), had lower proportion of drinking habits (P < 0.001), had higher prevalence of slow gait (P < 0.001), living alone (P = 0.003), and physical inactivity (P < 0.001), and had lower MMSE scores (P = 0.001) (Additional file 1: Table S3). In the Kaplan–Meier log-rank test, participants who scored ≥ 5 points in STAD at baseline had a significantly higher risk of dementia incidence than those who scored ≤ 4 points (*P* < 0.001, Fig. 2). Cox regression analysis showed that the HR (CI) for the incidence of dementia in participants who scored ≥ 5 points in STAD was 4.98 (2.61–9.48) in the crude model and 2.67 (1.40–5.08) in the adjusted model, including all potential confounding factors (age, sex, education, hypertension, heart disease, diabetes mellitus, hyperlipidemia, drinking habit, smoking habit, slow gait speed, living alone, physical inactivity, and cognitive function at baseline (Table 3).

**Table 3:** Hazard ratios and 95% CI for dementia incidence according to the STAD cut-off points

	Crude model		Adjusted model	
	HR	95% CI	HR	95% CI
STAD total score				
≤ 4 points	Reference		Reference	
≥ 5 points	4.98	2.61–9.48	2.67	1.40–5.08
Potential confounding factors				
Age (years)			1.10	1.05–1.16
Sex (female)			1.72	0.87–3.42
Education (years)			1.07	0.95–1.20
Hypertension (yes)			0.61	0.34–1.10
Heart disease (yes)			1.69	0.88–3.26
Diabetes mellitus (yes)			0.89	0.35–2.29
Hyperlipidemia (yes)			0.82	0.44–1.51
Drinking habit (yes)			1.27	0.69–2.33
Smoking habit (yes)			1.24	0.42–3.70
Slow gait speed (yes)			1.01	0.45–2.26
Living alone (yes)			1.14	0.46–2.83
Physical inactivity (yes)			0.96	0.51–1.82
MMSE (score)			0.83	0.74–0.94

The model was adjusted for all potential confounding factors assessed in the present study

*STAD* simplified telephone assessment for dementia risk, *HR* hazard ratio, *CI* confidence interval, *MMSE* mini-mental state examination

### Decision-tree analysis using STAD

**Fig. 3:**
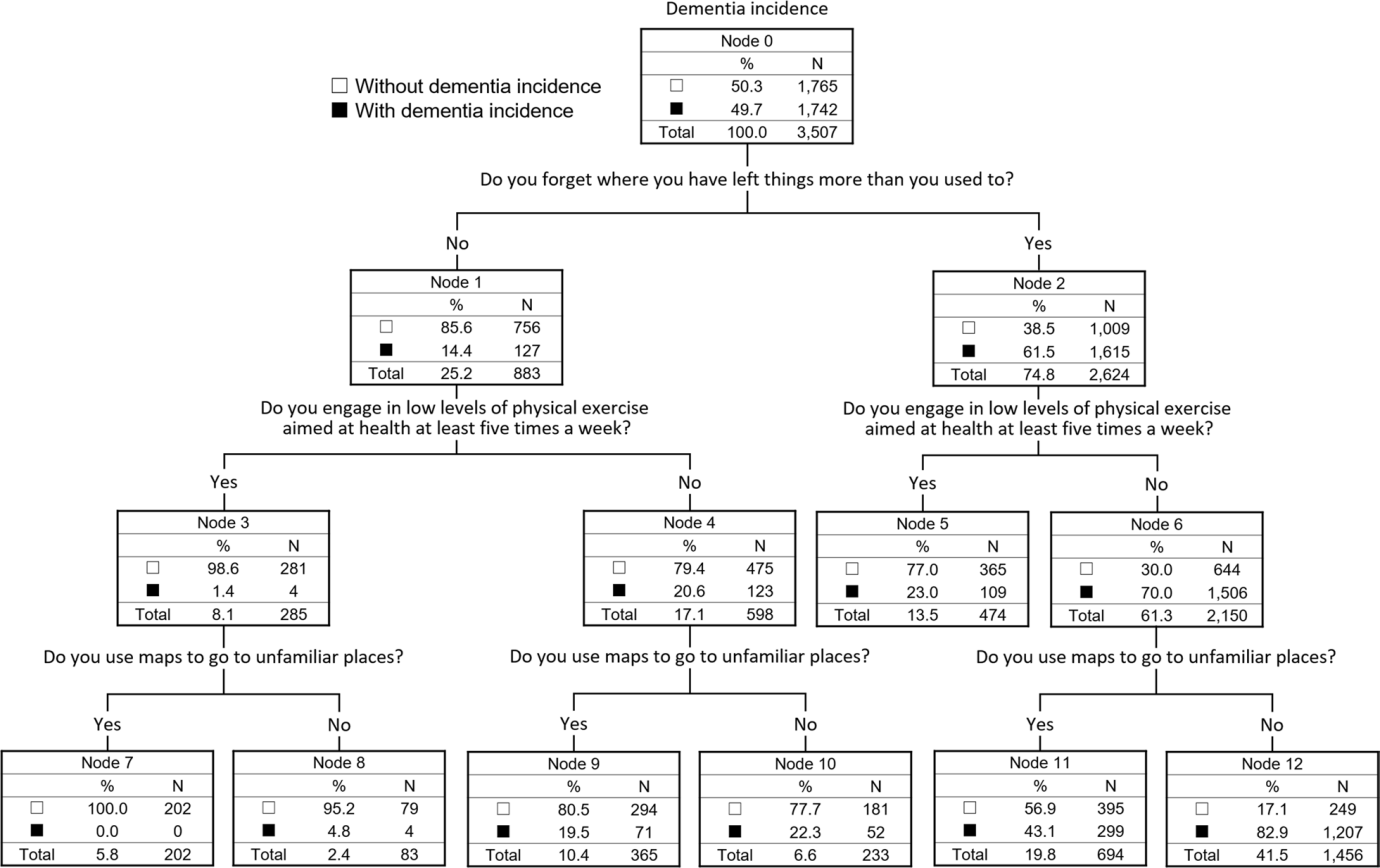
Decision tree model using STAD for the prediction of dementia

The final decision-tree model included 12 nodes with three items as follows: Do you forget where you have left things more than you used to? Do you engage in low levels of physical exercise aimed at health at least five times a week? Do you use maps to go to unfamiliar places? (Fig. 3). The CART subdivided the samples into seven risk groups with probabilities of dementia incidence ranging from 0.0 to 82.9%. The accuracy, sensitivity, specificity, and AUC were 0.65, 0.67, 0.65, and 0.70, respectively, in the logistic regression model and 0.83, 0.28, 0.85, and 0.65, respectively, in the decision-tree (CART) model (Table 4).

**Table 4:** Model performance of logistic regression and decision tree for dementia prediction in the test dataset

Models	Accuracy	Sensitivity	Specificity	AUC
Logistic regression	0.65	0.67	0.65	0.70
Decision tree (CART)	0.83	0.28	0.85	0.65

*CART* classification and regression tree, *AUC* area under the curve

## Discussion

We aimed to develop a screening tool to predict the risk of dementia that utilizes telephonic interviews and examined the predictive validity of the tool using a basic regression model and a decision-tree model. We developed a 12-item STAD questionnaire, and content validity was determined by experts. Our longitudinal analysis showed that cut-off points of the STAD score were significantly associated with the incidence of dementia over 24 months after adjusting for the covariates. Compared with the logistic regression model, the decision-tree model using the CART algorithm with three STAD items showed better predictive performance in terms of accuracy and specificity.

Regarding the development of STAD, we selected STAD items from literature review, statistical analysis with dementia incidence, and judgment of content validity by experts, resulting in 12 binary questions on subjective cognitive complaints, depressive symptoms, and lifestyle activities. Previous studies have demonstrated that some simplified questionnaires to assess subjective cognitive complaints [5], depressive symptoms [6], and lifestyle activities [7] could predict the risk of dementia from longitudinal analyses, and our results are in accordance with those findings. Moreover, all items of STAD were simplified “yes”/“no” questions, and we did not include questions to test cognitive functions directly; therefore, STAD must be acceptable in the first telephonic interview. Ortiz et al. reported that in cognitively impaired older adults, approximately half of them delayed or withheld diagnosis or treatment for over 18 months from their first symptoms, and most common barriers to healthcare access were personal beliefs [29]. For the timely diagnosis and treatment of dementia, STAD with a low-burden and easy-to-answer questionnaire is particularly useful for older adults with negative attitudes toward participation in health checkups or clinical examinations.

In the validation of STAD, optimal cut-off points (4/5 points) calculated based on the Youden index were significantly associated with new incidence of dementia over 24 months, after adjusting for covariates (including baseline MMSE score). Our results suggest that a combination of simple questions without biomarkers or neurocognitive tests can predict dementia risk. Furthermore, our decision-tree model using the CART algorithm showed that only three binary questions could predict the risk of dementia with certain accuracy. In the previous studies, we identified a few articles that have examined the usefulness of predictive models, including non-biological risk factors for dementia, using a decision-tree analysis [11–13]. Bang et al. and Dallora et al. proposed that decision-tree models consist of demographic or lifestyle activities, and they confirmed its’ certain predictive abilities [11, 12]. However, their predictive models included at least parts of the actual measurement values, such as performance of cognitive tests [11] or physical tests [12]; therefore, there were certain limitations in their application range. Li et al. reported the algorithmic association between easily obtainable non-biological risk factors (assessed by simplified questionnaire) in middle-age and dementia status [13], however, the usefulness of the decision-tree model for dementia prediction in older adults was unknown. Our results demonstrated usefulness of decision tree model that consists of only simplified questionnaires for dementia prediction among older adults. Particularly, our decision-tree model demonstrated a relatively low risk of false-positive results; thus, the STAD may be suitable for primary screening. Additionally, the decision-tree model using the CART algorithm provides an intuitive diagram to represent risk prediction without expert judgment; therefore, STAD is a useful tool in the population-based and non-face-to-face screening of dementia risk in community settings. Further, STAD may be applicable for subjects who are non-adherent to face-to-face assessments in long-term surveys or interventional studies. Encouragement of medical consultation based on STAD could improve the follow-up rate of outcomes, such as dementia incidence, in the above research field. Regarding each item of STAD adopted for the decision-tree model in our study, three questions on subjective cognitive complaints (Do you forget where you have left things more than you used to?), physical activity (Do you engage in low levels of physical exercise aimed at health at least five times a week?), and instrumental activity of daily living (Do you use maps to go to unfamiliar places?) were selected in the CART algorithm. Regular physical activity is a common protective factor for dementia in older people [30], and a recent meta-analysis confirmed a dose–response relationship between physical activity and dementia risk [31]. Exercising at least five times a week could be considered sufficient physical activity for older adults. Additionally, a previous study demonstrated that simple questions regarding the performance of instrumental activity of daily living can detect the earliest cognitive changes in clinically normal elderly individuals [32]. Moreover, using maps is related to going-out behaviors that comprise both physical and cognitive activities. This may be the reason our decision-tree model selected the STAD item related to not only subjective cognitive complaints but also physical and instrumental activities. Our findings suggest the importance of paying attention to changes in physical or instrumental activities of daily living as well as cognitive symptoms. Although this study developed dementia risk prediction models using only STAD items focusing on usability in telephonic interviews, further studies examining the possibility of improvement using additional variables that could be assessed by telephonic interviews are required.

A major strength of this study was that we analyzed well-characterized cohort data, including monthly follow-up of dementia, and conducted multivariable analyses after adjusting for multiple confounding factors. Additionally, we created a white-box decision-tree model without complicated calculations; thus, it could be implemented in real-world practice. However, the limitations of this study should be addressed. First, while we used the training and test data independently; our results should be further validated in other external cohorts that have similar characteristics. Second, our definition of dementia was only based on medical records according to the public health insurance systems, and the incidence rate of dementia could be, therefore, underestimated. In addition, we included the participants who were able to confirm public health insurance affiliations based on the Japanese National Health Insurance or Later-Stage Medical Care Systems; therefore, the participants who had Employees’ Health Insurance were excluded in this study. Third, we used an established statistical method (SMOTE) to solve the gap of sample size between participants with and without dementia. However, the imbalance of the original data might affect our results and this point should be taken into consideration when interpreting our findings. Fourth, the STAD is a subjective screening tool with neither informant interviews nor objective tests. Therefore, it should be utilized in primary screening in community settings rather than clinical settings. Finally, our sample came from a single cohort; therefore, further research examining cross-cultural validity of the STAD using diverse populations is required.

## Conclusion

We developed a 12-item questionnaire STAD as a screening tool to predict dementia risk utilizing telephonic interviews and confirmed predictive validity of the questionnaire for dementia incidence using a basic regression model and a decision-tree model. Our findings might provide useful information for the early screening of dementia risk and enable bridging between community and clinical settings. Additionally, STAD could be implemented without face-to-face meetings in a short time; therefore, it may be especially suitable for community-dwelling older adults who have negative attitudes towards clinical examination or are non-adherent to follow-up assessments in clinical trials.

## Supplementary Information

**Additional file 1: Table S1.** Adjusted hazard ratios and 95% confidence intervals for dementia incidence according to 30 binary questions. **Table S2.** Content validity index of 23 candidate items for STAD. **Table S3.** Baseline characteristics according to the STAD score in the test dataset.

## Data Availability

The datasets used and/or analyzed during the current study are available from the corresponding author on reasonable request.

## Abbreviations

STAD: Simplified telephone assessment for dementia risk
CART: Classification and regression tree
NCGG-SGS: National Center for Geriatrics and Gerontology-Study of Geriatric Syndromes
LTCI: Long-term care insurance system
MMSE: Mini-Mental State Examination
CVI: Content validity index
