# Supplementary material for: Development and validation of new screening tool for predicting dementia risk in community-dwelling older Japanese adults
Source: J Transl Med. 2021 Oct 26;19:448. doi: 10.1186/s12967-021-03121-9 (PMC8549197; doi:10.1186/s12967-021-03121-9)
Supplement: Supplementary file 1 — Additional file 1: Table S1. Adjusted hazard ratios and 95% confidence intervals for dementia incidence according to 30 binary questions. Table S2. Content validity index of 23 candidate items for STAD. Table S3. Baseline characteristics according to the STAD score in the test dataset. [file 12967_2021_3121_MOESM1_ESM.docx]

| **Table S1**. Adjusted hazard ratios and 95% confidence intervals for dementia incidence according to 30 binary questions | | | |
| --- | --- | --- | --- |
|  | | HR | 95% CI |
| Do you feel you have more problem with memory than most? | (yes) | 1.95 | 1.43-2.65 |
| Do you have any difficulty with your memory? | (yes) | 1.82 | 1.31-2.52 |
| Do you forget where you have left things more than you used to? | (yes) | 1.60 | 1.15-2.22 |
| Do you forget the names of close friends or relatives? | (yes) | 1.03 | 0.73-1.47 |
| Do other people find you forgetful? | (yes) | 1.83 | 1.31-2.55 |
| Do you find yourself not knowing today’s date? | (yes) | 1.63 | 1.18-2.24 |
| Have you dropped many of your activities and interests? | (yes) | 1.43 | 1.04-1.99 |
| Do you often get bored? | (yes) | 1.63 | 1.13-2.35 |
| Do you feel helpless? | (yes) | 1.49 | 1.09-2.04 |
| Do you feel you are helpful to friends or family? | (no) | 1.63 | 1.11-2.40 |
| Do you prefer to stay at home, rather than going out and doing new things? | (yes) | 1.63 | 1.20-2.23 |
| In the last 2 weeks have you felt tired without a reason? | (yes) | 1.46 | 1.00-2.14 |
| Have you lost 2 kg or more in the past 6 months? | (yes) | 2.08 | 1.48-2.93 |
| Have you experienced a fall in the past year? | (yes) | 1.44 | 1.01-2.05 |
| Are you afraid of falling? | (yes) | 1.62 | 1.17-2.26 |
| Do you avoid activities in daily life because of pain? | (yes) | 1.67 | 1.12-2.50 |
| Do you have any difficulties eating tough foods compared to 6 months ago? | (yes) | 1.49 | 1.07-2.08 |
| Do you normally stand up from a chair without any aids? | (no) | 1.33 | 0.82-2.15 |
| Do you normally climb stairs without using handrail or wall for support? | (no) | 1.42 | 1.01-2.00 |
| Do you go out at least once a week? | (no) | 1.41 | 0.78-2.54 |
| Do you go out less frequently compared to last year? | (yes) | 1.75 | 1.22-2.50 |
| Do you engage in paid work? | (no) | 1.31 | 0.87-1.96 |
| Do you manage your own deposits and savings at the bank? | (no) | 1.41 | 0.89-2.25 |
| Do you engage in low levels of physical exercise aimed at health at least five times a week? | (no) | 1.70 | 1.20-2.41 |
| Do you engage in hobbies or sports activities? | (no) | 1.78 | 1.30-2.45 |
| Do you engage in fieldwork or gardening? | (no) | 1.74 | 1.27-2.38 |
| Do you make a call by looking up phone numbers? | (no) | 1.40 | 0.80-2.47 |
| Do you use maps to go to unfamiliar places? | (no) | 1.52 | 1.09-2.12 |
| Do you use a personal computer? | (no) | 1.04 | 0.71-1.52 |
| Do you engage in cognitive stimulation such as board games and learning? | (no) | 1.55 | 1.12-2.15 |
| STAD = Simplified Telephone Assessment for Dementia risk; HR = hazard ratio; CI = confidence interval.  The HRs were calculated adjusting for age and sex. | | | |

| **Table S2**. Content validity index of 23 candidate items for STAD | | | | | |
| --- | --- | --- | --- | --- | --- |
|  | CVI | | | | Interpretation |
|  | Clarity | Concreteness | Essentiality | Importance |  |
| Do you feel you have more problem with memory than most? | 0.80 | 0.40 | 0.80 | 0.80 | Eliminated |
| Do you have any difficulty with your memory? | 0.80 | 0.40 | 0.80 | 1.00 | Eliminated |
| Do you forget where you have left things more than you used to? | 1.00 | 1.00 | 1.00 | 1.00 | Appropriate |
| Do other people find you forgetful? | 1.00 | 1.00 | 1.00 | 1.00 | Appropriate |
| Do you find yourself not knowing today’s date? | 1.00 | 1.00 | 0.80 | 1.00 | Appropriate |
| Have you dropped many of your activities and interests? | 1.00 | 1.00 | 1.00 | 1.00 | Appropriate |
| Do you often get bored? | 1.00 | 1.00 | 1.00 | 1.00 | Appropriate |
| Do you feel helpless? | 0.80 | 0.80 | 0.80 | 1.00 | Appropriate |
| Do you feel you are helpful to friends or family? | 1.00 | 1.00 | 0.60 | 1.00 | Eliminated |
| Do you prefer to stay at home, rather than going out and doing new things? | 0.80 | 0.80 | 0.80 | 0.80 | Appropriate |
| In the last 2 weeks have you felt tired without a reason? | 1.00 | 1.00 | 1.00 | 1.00 | Appropriate |
| Have you lost 2 kg or more in the past 6 months? | 1.00 | 1.00 | 0.40 | 0.80 | Eliminated |
| Have you experienced a fall in the past year? | 1.00 | 1.00 | 0.60 | 0.60 | Eliminated |
| Are you afraid of falling? | 1.00 | 1.00 | 0.60 | 0.60 | Eliminated |
| Do you avoid activities in daily life because of pain? | 1.00 | 1.00 | 0.20 | 0.40 | Eliminated |
| Do you have any difficulties eating tough foods compared to 6 months ago? | 1.00 | 1.00 | 0.20 | 0.60 | Eliminated |
| Do you normally climb stairs without using handrail or wall for support? | 1.00 | 1.00 | 0.60 | 0.80 | Eliminated |
| Do you go out less frequently compared to last year? | 1.00 | 1.00 | 0.80 | 1.00 | Appropriate |
| Do you engage in low levels of physical exercise aimed at health at least five times a week? | 1.00 | 0.80 | 1.00 | 0.80 | Appropriate |
| Do you engage in hobbies or sports activities? | 0.80 | 0.80 | 0.60 | 1.00 | Eliminated |
| Do you engage in fieldwork or gardening? | 1.00 | 1.00 | 0.60 | 0.60 | Eliminated |
| Do you use maps to go to unfamiliar places? | 1.00 | 1.00 | 0.80 | 0.80 | Appropriate |
| Do you engage in cognitive stimulation such as board games and learning? | 1.00 | 1.00 | 1.00 | 1.00 | Appropriate |
| STAD = Simplified Telephone Assessment for Dementia risk; CVI = content validity index. | | | | | |

| **Table S3**. Baseline characteristics according to the STAD score in the test dataset. | | | | |
| --- | --- | --- | --- | --- |
|  | | Participants who scored  ≤4 points in STAD  *n* = 1,132 | Participants who scored  ≥5 points in STAD  *n* = 618 | *P-*value^*^ |
| Age | (years) | 71.4 ± 4.9 | 73.0 ± 6.0 | <0.001 |
| Sex | (female, %) | 519 (45.8) | 357 (57.8) | <0.001 |
| Education | (years) | 11.7 ± 2.5 | 10.8 ± 2.5 | <0.001 |
| Hypertension | (n, %) | 525 (46.4) | 306 (49.5) | 0.209 |
| Heart disease | (n, %) | 176 (15.5) | 106 (17.2) | 0.383 |
| Diabetes mellitus | (n, %) | 158 (14.0) | 98 (15.9) | 0.282 |
| Hyperlipidemia | (n, %) | 473 (41.8) | 254 (41.1) | 0.781 |
| Drinking habit | (n, %) | 563 (49.7) | 245 (39.6) | <0.001 |
| Smoking habit | (n, %) | 108 (9.5) | 51 (8.3) | 0.370 |
| Slow gait speed | (n, %) | 65 (5.7) | 89 (14.5) | <0.001 |
| Living alone | (n, %) | 85 (7.5) | 73 (11.8) | 0.003 |
| Physical inactivity | (n, %) | 261 (23.1) | 269 (43.5) | <0.001 |
| MMSE | (score) | 26.7 ± 2.3 | 26.3 ± 2.4 | 0.001 |
| MMSE, mini-mental state examination.  The data are expressed as the mean ± standard deviation or numbers (%).  **^*^**Based on Student’s t-test for continuous variables and χ^2^ tests for categorical variables. | | | | |
